# A 35-year-old woman presenting with an unusual post-traumatic leiomyoma of the nipple: a case report

**DOI:** 10.1186/1752-1947-7-49

**Published:** 2013-02-19

**Authors:** Leonidas Pavlidis, Efstratios Vakirlis, Georgia-Alexandra Spyropoulou, Manousos Georgios Pramateftakis, Dimitris Dionyssiou, Efterpi Demiri

**Affiliations:** 1 Plastic Surgery Department, Aristotle University of Thessaloniki, Papageorgiou Hospital Thessaloniki, Thessaloniki, Greece; 2 First Department of Dermatology and Venereology, Aristotle University of Thessaloniki, Delfon 124, Thessaloniki, Greece; 3 Department of Surgery, European Interbalkan Medical Center, Asklipiou 10, Pylaia, Thessaloniki, Greece

**Keywords:** Benign tumors, Nipple, Leiomyoma

## Abstract

**Introduction:**

Leiomyoma of the mammary papilla is one of the most uncommon nipple tumors with only 50 cases reported in the literature until now. To the best of our knowledge we present the first report of a nipple leiomyoma that originated from a traumatic abrasion caused by breastfeeding.

**Case presentation:**

A 35-year-old healthy Caucasian female with a cauliflower-like tender and pink nodular mass that was approximately 10mm in diameter presented to our out-patients department. The patient suggested that the mass originated from a traumatic abrasion caused by breastfeeding three years ago and it has been slowly growing ever since.

An excision biopsy was performed. The histological and immunohistochemical examination confirmed the diagnosis of leiomyoma. There were no postoperative complications or any sign of local recurrence four years postoperatively.

**Conclusions:**

Leiomyoma of the mammary papilla is a rare benign neoplasm that usually appears as a solid tender nodule. Differential diagnosis comprises breast carcinoma, leiomyosarcoma and myoid hamartoma. The recommended treatment is complete excision of the tumor with histologically confirmed tumor-free margins otherwise recurrence is possible. A detailed history of the patient’s disease can reveal the original etiology. This is an original case report that will have particular interest to plastic surgeons, dermatologists, and pathologists. The pathogenetic mechanism was trauma of the nipple. According to our review of the literature this particular information has never been reported and we think that it may advance our knowledge of this very infrequent tumor.

## Introduction

Leiomyoma of the mammary papilla is one of the most uncommon nipple-areola tumors. According to our review of the literature, 50 cases have been reported to date [1-10]. There are different types of leiomyomas depending on the tissue origin, such as vascular leiomyoma (from vascular endothelium) [2] or superficial leiomyoma (from dermal smooth muscle) [3]. The histogenesis of leiomyomas remains a matter of controversy [4]. We report a case of nipple leiomyoma that originated from a traumatic abrasion caused by breastfeeding.

## Case presentation

A 35-year-old Caucasian woman previously fit and well with a three-year history of a painful lump of the right breast closely associated anatomically to the nipple presented to our out-patients department.

The pain was quite severe, squeezing in nature, radiating to her nipple and deep into her breast and regular painkillers did not seem to alleviate the symptoms.

The lump had slowly increased substantially in size since it was first noted. The patient suggested that the mass originated from a traumatic abrasion caused by breastfeeding three years ago and it has been slowly growing ever since. Due to the unknown histogenesis of the tumor this abrasion could be the reason for its unilateral appearance.

The rest of the history revealed the patient to be a mother of twins, on contraceptives (ethinylestradiol and cyproterone acetate) and heavy smoker.

**Figure 1:**
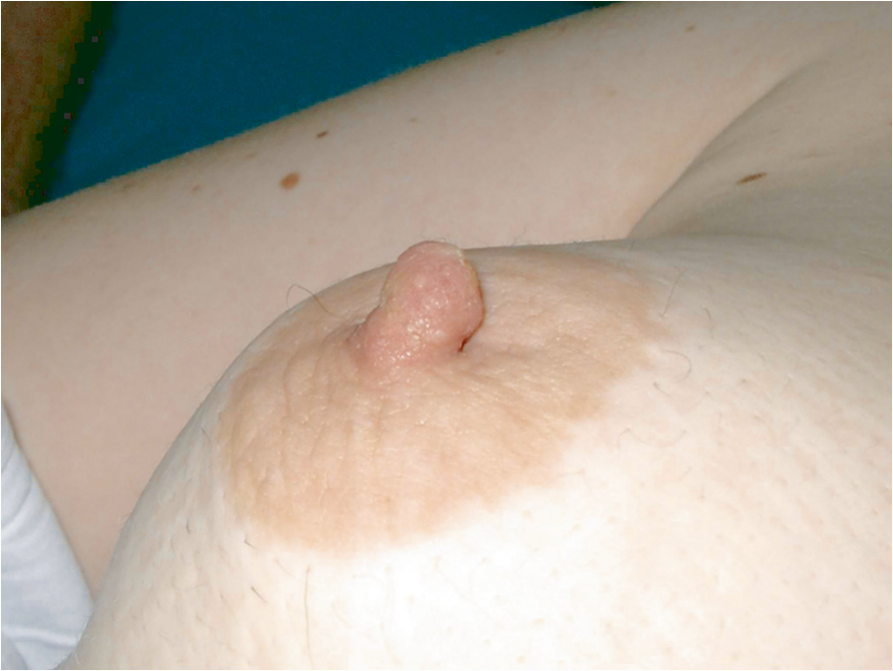
Nipple leiomyoma on the right breast.

A physical examination revealed a pink cauliflower-shaped tender lump of one cm in diameter (Figure 1) positioned just superiorly to the right nipple. The lesion was quite firm and appeared to be fixed to the underlying tissues. No discharge from the patient’s nipple was appreciated.

There was neither translumination nor associated lymphadenopathy. The patient had a mammogram performed preoperatively which showed bilateral dense breasts with minimal fatty replacement.

The patient underwent an excision biopsy and the pathology report revealed that the lesion was completely removed. Her nipple was preserved. Sutures were removed on the eighth day postoperatively. Pain disappeared immediately after the operation and never reappeared.

**Figure 2:**
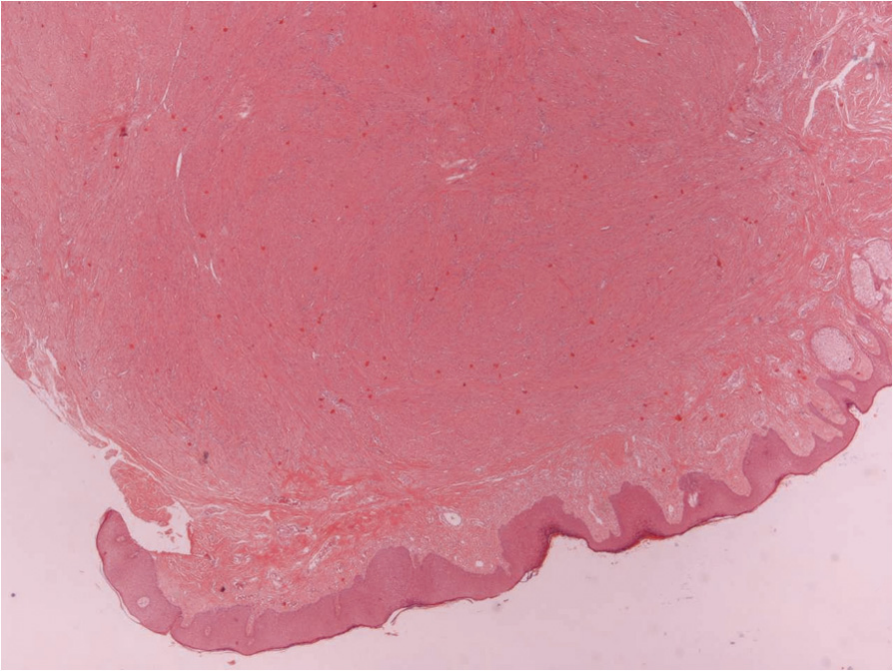
Skin section of the nipple with a well-circumscribed mesenchymal tumor in the dermis and underlying mammary gland (magnification × 20).

**Figure 3:**
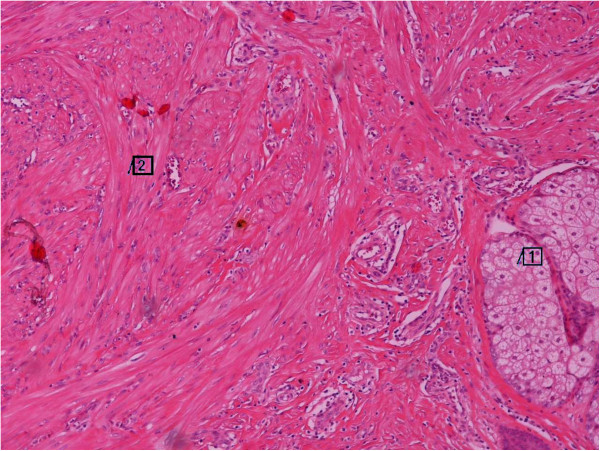
**The neoplasm composed of bundles of elongated cells with oval-shaped nuclei (insert 2).** The neoplasm infiltrating the upper dermis with adjacent epidermal appendages (insert 1) (magnification × 200).

**Figure 4:**
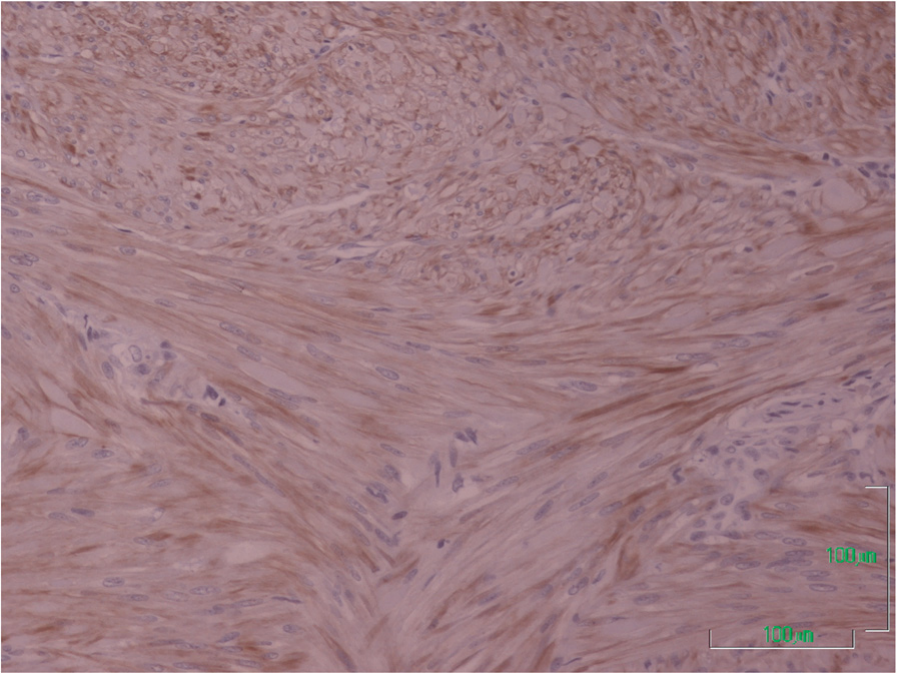
The neoplastic cells expressed cytoplasmic immunoreactivity for the smooth muscle actin antigen (cytoplasmic brown color, magnification × 400).

The histological examination showed a completely resected mammary subpapillary neoplasm, composed of interlacing bundles of smooth muscle cells separated by a small amount of well-vascularized connective tissue (Figure 2). The tumor was well circumscribed and involved the dermis and the adjacent mammary gland (Figure 3). The neoplastic cells were elongated spindle cells with oval-shaped nuclei and random mitoses. An immunohistochemical examination for smooth muscle actin (SMA), a marker for non-striated muscles, was also performed. The majority of neoplastic cells expressed a positive cytoplasmic immunoreactivity for SMA, indicating that the neoplasm is of smooth muscle origin (Figure 4).

Thus, the diagnosis of leiomyoma was confirmed. There were no postoperative complications or any sign of local recurrence four years postoperatively.

## Discussion

Leiomyoma of the mammary papilla is a rare benign neoplasm first described by Virchow in 1854 [6]. The cause or triggering or appearance mechanism of this neoplasm is unknown therefore an abrasion during breastfeeding could possibly have a relation to the appearance of the tumor. The main reason that leads patients with nipple leiomyomas to their physicians is chronic pain [11]. The lesion is benign but has a high rate of local recurrence that can reach up to 50%. The tumors are treated with either surgical resection or CO_2_ laser ablation and have low recurrence rates [12]. There have been no reports on a correlation with epithelial tumors or carcinomas [7], or with any genetic predisposition. Of interest, the right breast is more frequently involved but there has been no report of bilateral appearance of the tumor [8]. Leiomyoma of the nipple consists of interlacing bundles of smooth muscle fibers, with no or minimal intervening tissue. Glandular elements, necrosis, nuclear atypia or mitotic activity are absent within the lesion [2,9,10]. Immunohistochemically the tumor cells express desmin, SMA and vimentin but not S-100 or cytokeratin [13]. Leiomyoma of the nipple has recently been reported to be estrogen and progesterone receptor positive therefore the appearance and progression of the tumor might have a possible relation to the medical history of contraceptive administration to the patient [14].

The main differential diagnosis includes myoid hamartoma and especially leiomyosarcoma. Myoid hamartoma is a benign lesion with a histological picture of glandular elements scattered between the spindle cell bundles, and evidence of sclerosing adenosis at the edge of the lesion. Leiomyosarcoma is a malignant lesion that presents in patients between the ages of 50 and 75 years and may have slight male predominance [15]. By contrast, leiomyoma rarely presents in male patients [1]. Moreover, in leiomyosarcoma the lesion is more cellular with pleomorphism, mitotic activity and sometimes necrosis. According to our review of the literature this is the first report of leiomyoma of the nipple originating after trauma to the nipple during breastfeeding.

## Conclusions

Leiomyoma of the mammary papilla is a rare benign neoplasm which can be the result of a traumatic abrasion of the nipple. This tumor can cause chronic severe pain. Careful history taking and excision of the tumor with histologically confirmed tumor-free margins is the recommended treatment because there is a reported high risk of recurrence.

## Consent

Written informed consent was obtained from the patient for publication of this manuscript and any accompanying images. A copy of the written consent is available for review by the Editor-in-Chief of this journal.

## Competing interests

We declare that we have no competing interests.

## Authors’ contributions

LP and GS drafted the manuscript. MGP, EV and DD equally contributed to data collection and ED supervised manuscript writing. All authors read and approved the final manuscript.
